# Sense of coherence in family caregivers of children with chronic diseases: a narrative review

**DOI:** 10.3389/fped.2026.1820131

**Published:** 2026-05-07

**Authors:** Fangyu Shi, Junjie Zhao, Xin Zhang

**Affiliations:** College of Health Management, Shanghai Jian Qiao University, Shanghai, China

**Keywords:** sense of cohearence, child, chronic diseases, family caregivers, narrative review

## Abstract

In recent years, as the prevalence of chronic diseases in children continues to increase significantly in the world, family caregivers who undertake long-term care responsibilities are increasingly facing a huge psychological burden. As the core theoretical construction of positive psychology, Antonovsky's Sense of Coherence (SOC) has become an important psychological resource that enables caregivers to actively cope with the multiple stressors inherent in the care process and maintain their physical and mental balance. This article reviews the research status of sense of coherence in family caregivers of children with chronic diseases, including the theoretical connotation and evolution of sense of coherence, the psychometric characteristics of existing measurement tools, a variety of influencing factors, and the current mainstream intervention strategies. In addition, the review critically dissects controversies, methodological flaws, and inherent gaps in the current body of literature and suggests targeted directions for future research. This study aims to provide a solid theoretical and empirical basis for clinicians to formulate targeted intervention programs, so as to improve the mental health status of family caregivers and optimize the overall care system for children with chronic diseases.

## 1 Introduction

A chronic disease in children is defined as a condition characterized by persistent symptoms lasting for more than 3 months or recurrent episodes occurring at least three times within a recent period (1). The most common chronic childhood diseases are asthma, obesity, diabetes, as well as mental illness and developmental disabilities (2). In recent decades, the global incidence of chronic diseases in children has surged dramatically, evolving into a pressing public health concern that contributes substantially to childhood morbidity, mortality, and long-term disease burdens. In 1960, a mere 1.8% of children worldwide were living with a chronic medical condition; by 2010, the proportion had climbed to 8%,and current projections suggest a continued upward trajectory driven by factors such as changing lifestyles, environmental influences, and improved diagnostic capabilities (3). The diagnosis and treatment of chronic diseases in children is a stressful process for both children and their parents, often disrupting the entire family system's functional dynamics (4). In most cases, the primary responsibility for daily caregiving falls to parents, who must navigate the complexities of managing their child's medical needs while balancing competing demands such as other parental obligations, career responsibilities, social relationships, and personal aspirations. When the child is diagnosed with chronic diseases, the caregiver may have to accompany the child or even face the loss of the child. Such long-term, high-intensity, and emotionally taxing caregiving roles render caregivers particularly vulnerable to a range of adverse psychological outcomes, including anxiety, depression, burnout, and diminished quality of life (5–7).

**Figure 1:**
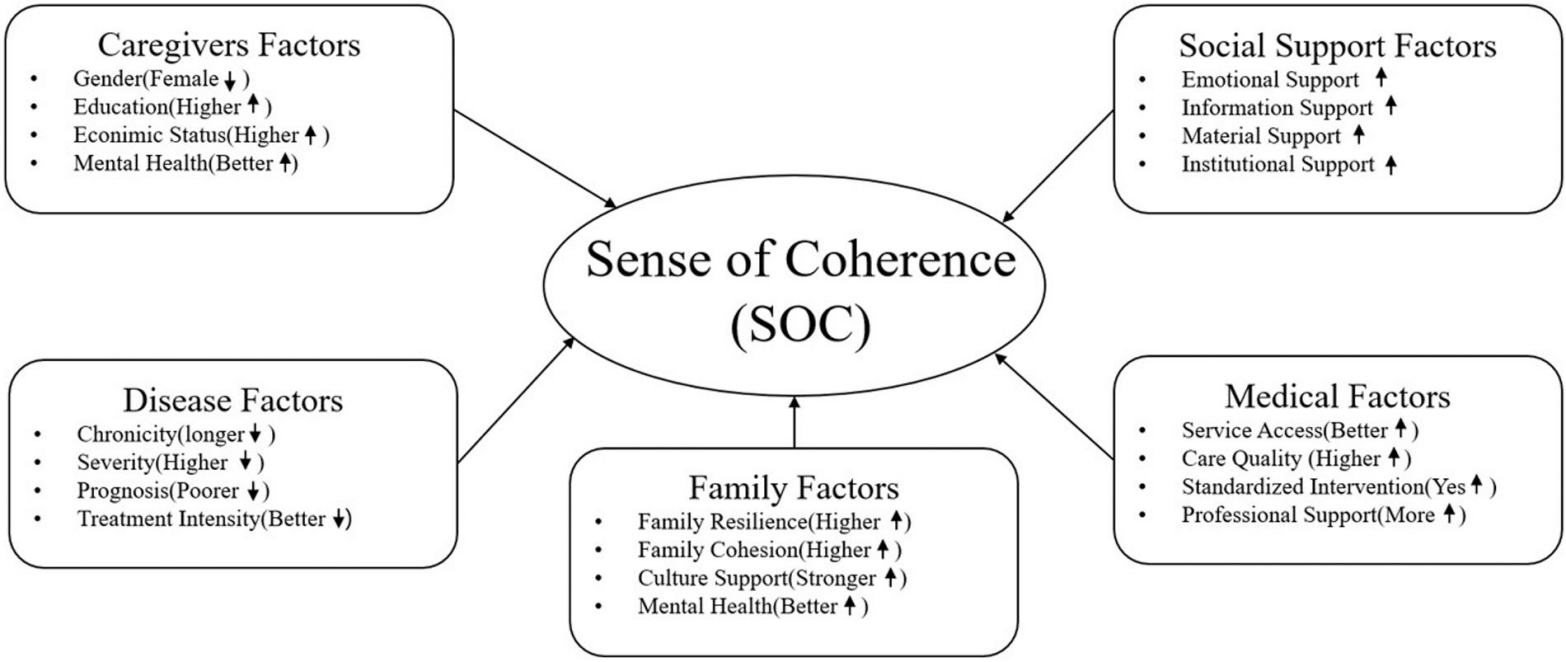
Mechanism of factors influencing sense of coherence(SOC).

**Table 1:** Comparative analysis of measurement tools.

Scale Name	Number of Items	Scoring Method	Total Score Range	Cronbach's *α*	Advantages	Disadvantages
SOC-29 Scale (10)	29	7-point score (1–7 points per question)	29–203	0.70–0.95	Good cross-cultural adaptability and It has been translated into 32 languages.	Too many items, so it is not suitable for large-scale population testing. It needs to be localized.
SOC-13 Scale (15)	13	7 points (1–7 points, 5 inverse scores)	13–91	0.70–0.92	Items are short and widely used in Finland, Japan, Brazil, China, Peru, Australia and so on.	Its 7-point rating system may lead to a higher coefficient of variation, affecting the reliability of the scale.
SOC-L9 Scale (16)	9	7 points score (1–7 points, 4 reverse scores)	9–63	0.732	Testing time is short, suitable for large-scale surveys or clinical screenings.	Only one dimension to test, lower reliability,with limited applicability for screenings.

The advent of positive psychology provides a new perspective for the research of caregivers' mental health, among which Antonovsky's (SOC) theory has gradually become the core research direction. SOC is defined as the positive belief that individuals have that they can effectively cope with internal and external environmental pressures and use resources to maintain physical and mental health (8). Previous studies have confirmed that high-level SOC can help caregivers better manage care burden, buffer the negative effects of stress on physical and mental health, and improve quality of life (9). However, the current research on SOC of family caregivers of children is still in its infancy, and the depth and breadth of research are insufficient, which is difficult to meet the needs of clinical practice. Specifically, existing studies often lack methodological diversity, fail to capture the dynamic nature of SOC over time, and inadequately address the unique needs of underrepresented caregiver populations. Based on the current research, this review aims to consolidate the current state of knowledge on SOC in this vulnerable group, identify critical research gaps, and offer actionable recommendations to inform future research and clinical intervention strategies.

## 2 Theoretical connotation and development of sense of coherence (SOC)

SOC theory originates from Antonovsky's salutogenesis theory, first proposed in 1979, which broke through the traditional “disease-oriented” research paradigm and instead focused on “how health is generated and maintained”. 8 Antonovsky believed that health is not a static state of “disease-free”, but a dynamic process in which individuals constantly adapt to the stressful environment and maintain physical and mental balance, and SOC is the core psychological mechanism of this process (10). It is defined as an individual's overall perception of the understandability of the environment, the manageability of resources and the sense of meaning in coping with stress.

SOC comprises three interdependent dimensions; these three dimensions are interrelated and have dynamic effects.

- **Comprehensibility**: “Comprehensibility” is the cognitive basis, and caregivers can clearly recognize the characteristics of children's diseases, care needs and sources of pressure.
- **Manageability**: The belief that caregivers perceive that they have sufficient material, emotional, or social resources to cope with care challenges.
- **Meaningfulness**: “Sense of meaning” is considered to be the core of sense of coherence, which refers to whether caregivers can feel value and meaning from the care process, rather than a simple burden.

These three dimensions form a “psychological protection mechanism” that enables caregivers to navigate the complexities of chronic caregiving with resilience and adaptive coping strategies. A high level of SOC is thus associated with a greater likelihood of caregivers adapting positively to their caregiving role, maintaining the physical and mental health of themselves and children (11).

In 1987, Antonovsky further proposed the “ Healthy Model”on the basis of health origin theory, which extended the application of SOC from the individual level to broader social systems, including families, communities, and organizations.It emphasized the health principle of “active compliance”—that is, individuals should not only reduce risk factors, but also proactively seek out and utilize internal and external resources (11). This model provides a theoretical framework for the study of SOC of family caregivers of children: SOC of caregivers is not only affected by their own psychological characteristics, but also closely related to external factors such as family support and social resources. It was not until the early 2000s that researchers began to extend SOC research to caregiver populations, recognizing its potential as a key resilience factor in mitigating caregiving burden. In 2002, a study specifically examining SOC among parents of children with autistic spectrum disorders(ASD) was conducted by Bengt Sivberg, who compared the SOC levels of parents of children with ASD and parents of non-autistic children. The findings revealed that the SOC levels of parents of children with ASD were significantly lower (12). This landmark study laid the groundwork for subsequent research, highlighting the relevance of SOC to the unique challenges faced by this caregiver population and underscoring the need for targeted interventions to enhance SOC.

## 3 SOC status of family caregivers of pediatric patients

Based on the current research data on the sense of coherence (SOC) of family caregivers of children with chronic diseases, the level of SOC of family caregivers of children with chronic diseases is generally lower than that of caregivers of healthy children. This finding is robust across diverse cultural contexts and disease populations, reflecting the unique stressors inherent in chronic pediatric caregiving.

A cross-sectional study of 287 family caregivers of children with acute leukemia in China showed that the level of sense of coherence of caregivers of children with acute leukemia was significantly lower. The researchers found that the average total SOC score among caregivers was 57.57 ± 11.14. While this score exceeds the midpoint of the scale's range (13–91) points; midpoint = 52 points), it falls within the low-level range (≤63 points) (13), indicating that the majority of caregivers in this sample experienced significant challenges in perceiving their caregiving situation as comprehensible, manageable, or meaningful. Complementary evidence comes from a systematic mixed-methods review conducted in the United States, which explored the psychological experiences of parents of children with type 1 diabetes. The review found that 33.5% of parents reported significant psychological distress at the time of their children's diagnosis, and 19% continued to experience persistent distress 1 to 4 years post-diagnosis. Although parents would gradually adapt to this distress, this prolonged exposure to stress was found to be directly associated with lower SOC levels (14). Furthermore, the study identified a significant negative correlation between parental SOC and child treatment non-adherence, highlighting the broader implications of caregiver SOC for child health outcomes.

## 4 Measurement tools for SOC

### 4.1 SOC-29 scale

It was developed by Antonovsky in 1987 and is the earliest SOC measurement tool. It comprises 3 dimensions: comprehensibility (11 items), manageability (10 items) and meaningfulness (8 items), with a total of 29 items. The scale was scored on a 7-point Likert scale (1 = “not at all” to 7 = “completely”), with total scores ranging from 29 to 203, where higher scores indicate higher SOC levels. The SOC-29 Scale has demonstrated excellent psychometric properties, with Cronbach's *α* coefficients ranging from 0.82 to 0.95, indicating high internal consistency (10). It was initially applied to the general population, and then gradually applied to the caregivers of patients. However, its utility in research involving family caregivers of children with chronic diseases is limited by its length: completing the scale typically requires 15 to 20 min,which may lead to a low response rate or random answers due to busy care tasks, compromising the validity of the data. Consequently, it is rarely used in the research of caregivers of children.

### 4.2 SOC-13 scale

In order to solve the problem of lengthy items in SOC-29, Antonovsky selected 13 core items from SOC-29 and formed a simplified version of SOC-13, which still retained 3 dimensions (5 items of intelligible sense, 4 items of manageable sense, and 4 items of meaning sense). The SOC-13 scale has demonstrated strong psychometric properties, with Cronbach's *α* coefficients ranging from 0.70 to 0.92 and a test-retest reliability ranging from 0.55 to 0.75, indicating good temporal stability over time. Its primary advantage lies in its brevity: completing the scale takes only 5 to 10 min, making it more feasible for use with busy caregivers. Due to its brevity (5 to 10 min to complete) and good psychometric properties, SOC-13 has become the most commonly used tool in SOC research of caregivers of children (15). However, the scale is not without limitations: the 7-point response format can lead to large variability in scores, and some studies have questioned the structural validity of certain items, particularly in cross-cultural contexts.

### 4.3 SOC-L9 scale

The SOC-L9 Scale uses a 7-point Likert response format, with total scores ranging from 9 to 63, and has demonstrated acceptable internal consistency (Cronbach's *α* = 0.87). The scale's unidimensional structure enhances its cross-cultural adaptability, as it minimizes the potential for cultural differences in the interpretation of a multidimensional construct. However, its utility is limited by its lack of dimensional specificity: by combining the three dimensions into one, the SOC-L9 scale cannot provide insights into which specific aspects of SOC may be most compromised or in need of intervention (16). Consequently, it is primarily used in studies where a brief, global measure of SOC is sufficient, rather than those requiring a detailed analysis of dimensional differences. Comparison of measurement tools is in Table 1.

## 5 Influencing factors of SOC in family caregivers of pediatric patients

### 5.1 Caregiver-related factors

#### 5.1.1 Gender

Studies have shown that female caregivers have significantly lower SOC levels than men (17). This gender-based difference may be attributed to a combination of biological, psychological, and sociocultural factors. Women tend to be more emotionally sensitive and attuned to nuanced emotional cues than men, whereas men tend to adopt a more problem-focused and rational coping style, which may buffer against stress. Studies of parents of children with ADHD have shown that mothers are more likely to be worried and fathers are more likely to adopt a “wait-and-see” strategy (18, 19), and other studies have shown that mothers who take more responsibility for child follow-up are more stressed in families of children with ADHD (20). A UN Women survey in Europe and Central Asia showed that 70% of women perform at least one type of domestic work, which was 11% points higher than that of men, and the proportion of women who took on additional care responsibilities was 9% points higher than that of men (21). This gender division of labor further increased the burden of care on female caregivers and affected SOC levels more significantly. While previous research on caregivers of children with autism has similarly confirmed this idea, mothers may bear more stress and thus may have a lower level of sense of coherence (22–24). The role of fathers cannot be ignored. A survey of parents of children diagnosed with type 1 diabetes highlighted the need to involve fathers in treatment and support after their adolescent child is diagnosed, and to strengthen the unique role of fathers in the parent-child relationship (25). However, findings from another study of parents of children with autism failed to confirm such differences in the level of sense of coherence between mothers and fathers in groups of parents of children with autism or groups of parents of typically developing children (26). In addition, a longitudinal study of parents of children with cancer, conducted by Ingrid Bergh et al., showed that parents' sense of coherence levels dynamically changed over the course of their children's treatment, with fathers' scores decreasing later in childhood cancer treatment, while mothers' scores had already decreased (27). These conflicting findings highlight the need for further research to explore the contextual factors that moderate the relationship between gender and SOC, such as cultural norms, family support, and the nature of the child's disease.

#### 5.1.2 Education level

Educational level is strongly positively correlated with caregiver SOC: caregivers with higher educational attainment tend to report stronger SOC than those with lower educational attainment. This relationship may be explained by several mechanisms. First, individuals with higher education are more likely to possess the cognitive skills and health literacy necessary to understand complex medical information, navigate healthcare systems, and make informed decisions about their child's care, enhancing their sense of comprehensibility. Second, higher education is often associated with greater access to social and economic resources, which can improve caregivers' sense of manageability. Third, individuals with higher education are more likely to possess the cognitive skills and health literacy skills necessary to understand complex medical information, which can foster a stronger sense of meaningfulness.

Studies of caregivers of children with ASD in eastern China have also confirmed that caregivers with high school education or above have higher quality of life and may also have relatively stronger SOC levels (28). Some studies have further pointed out that caregivers with low education levels also face the problem of limited access to disease knowledge, especially in remote areas. Due to the lack of localized disease science resources, even if they have the intention to obtain medical information, it is difficult to translate it into care ability, further widening the SOC gap between caregivers with high education levels (29).

#### 5.1.3 Economic conditions

Economic status is another significant reason of caregiver SOC, with higher family income consistently associated with stronger SOC. Financial stability provides caregivers with access to better medical care, specialized treatments, and additional support services (such as paid caregivers or respite care), which can reduce caregiving burden and enhance the sanse of manageability.A study of caregivers of children with cancer showed that higher-income families had access to better medical services and reduced anxiety caused by financial pressure (30). At the same time, high-income families can hire caregivers to assist in care, reduce the burden of caregivers, and improve the sense of manageability. Chinese studies on families of children with WS have also pointed out that perceived economic burden is an independent factor affecting the quality of family life, and long-term economic pressure can deplete psychological resources of caregivers, leading to a decrease in their sense of control and meaning in life, and thus a decline in their SOC levels (31). For countries with less developed economies, the impact of economic conditions is more prominent. Some low-income families in Uzbekistan and other countries need to interrupt treatment frequently because they can not afford the medical expenses of their children, which significantly reduces the sense of management and meaning of caregivers. Families participating in the national medical mutual assistance program have a significantly higher SOC level of caregivers (32). These findings underscore the importance of addressing economic barriers to care through policy interventions such as expanded health insurance coverage, financial assistance programs, and subsidies for caregiving services.

#### 5.1.4 Psychological factors

The mental health status of caregivers directly affects SOC. This relationship is bidirectional: a high SOC can protect caregivers from developing mental health problems, while poor mental health can gradually erode SOC levels over time. Gugala's study on caregivers of children with cerebral palsy found that the SOC level of caregivers without anxiety and depression symptoms was 12.3 points higher than those of caregivers with such symptoms. Mentally healthy caregivers are more likely to rationally understand their children's medical conditions, reduce the fear of “unknown risks”, and improve the sense of understanding. At the same time, they were more willing to seek support from family and friends, and had a stronger sense of manageability and meaning. Conversely, negative emotions will form a vicious circle of “stress-emotional deterioration -SOC decline”. Caregivers have difficulty understanding disease knowledge due to anxiety, and lack of motivation to care due to depression, which eventually leads to a continuous decrease in SOC level (33). Another study of mothers of children with autism found that depressed mothers of children with autism had consistently lower levels of sense of coherence (34). Additionally,a study of parents of children with intellectual disabilities also found that lower SOC was associated with poor parental mental health and that such parents were more likely to use physical punishment against their children (35), highlighting the potential cascading effects of low SOC on both caregiver and child outcomes. To break this cycle, interventions should prioritize the mental health of caregivers, integrating screening and treatment for anxiety and depression into routine care.

### 5.2 Disease factors

The disease of children is also an important factor affecting the SOC of caregivers. The chronicity of the child's diseases also influences the SOC level of caregivers. Diseases that require long-term treatment, such as chronic kidney disease and congenital heart disease, will lead to long-term high pressure in caregivers, imposing sustained caregiving demands that can gradually erode caregiver SOC in the absence of effective support.In the absence of effective intervention, SOC level tend to decline as the care period lengthens. Studies have shown that the SOC level of caregivers of children with congenital heart disease is directly related to the effect of national medical intervention. In families of children receiving standardized treatment, the SOC level of caregivers gradually increases with the stability of the children's condition (36). Another study showed that the average SOC score of family caregivers of children with leukemia was 57.57 ± 11.14, which was lower than that of parents of children with autism (59.91 ± 11.04), but higher than that of parents of children with cancer (51.40 ± 14.20) (13, 37, 38). These differences may reflect variations in disease prognosis, treatment intensity, and the level of uncertainty associated with each condition.

### 5.3 Family factors

Family resilience,defined as the ability of a family to adapt and thrive in the face of adversity, is a key protective factor for caregiver SOC. A cross-sectional study of 205 caregivers of children with autism found that family resilience played a significant mediating role between care burden and SOC. Good family resilience could buffer the negative effects of care burden and help caregivers reduce stress in the process of caring for children (39). Suparit's qualitative research on caregivers of children with leukemia also found that caregivers with strong family resilience were more likely to find “positive meaning” from the crisis, such as strengthened family bonds or personal growth, which enhanced their sense of meaningfulness (40). The family resilience in Central Asian countries is significantly affected by traditional culture. Kazakstan families follow the principle of “Zheti ata” (knowing the ancestors of seven generations before). This cultural value encourages family members to collaborate in caregiving, reducing the burden on the primary caregiver and enhancing their sense of manageability.

Family cohesion, defined as the emotional bonding and closeness among family members, is another critical factor that enables family members to collaborate in caring for child, and the sense of management of caregivers is significantly higher than that of families without family support (41). At the same time, its tradition of mutual assistance also enhances resilience, and the sharing of care among relatives effectively reduces the burden on the primary caregiver. Indonesian parents of children with disabilities embrace self-reliance and religious/spiritual dimensions of coping attitudes and strive to cope with psychological difficulties by valuing family relationships and obtaining support (42). On the other hand, family cohesion directly affects the SOC level of caregivers. Studies on families of children with ASD have shown that a high cohesive family can provide emotional support and a collaborative environment for caregivers, reduce the sense of isolation, and thus improve their SOC level (43).

To enhance caregiver SOC, interventions should focus on strengthening family resilience and cohesion. This may include family therapy, support groups for families of children with chronic diseases, and educational programs that teach families effective communication and coping skills. Additionally, cultural values that promote family support and mutual assistance should be leveraged to develop contextually appropriate interventions.

### 5.4 Medical factors

The accessibility and quality of medical care are critical reasons of caregiver SOC, as they directly influence caregivers’ perceptions of their child's care and their ability to cope with caregiving demands. Studies have found that the rational use of paid nursing services can effectively alleviate the care burden of caregivers in specific dimensions, such as daily care pressure and psychological pressure, and the reduction of burden helps to maintain and improve SOC (44). Caregivers with higher perceived quality of medical services have better QOL, and behind this association may be that professional medical support reduces care uncertainty and enhances caregiver coping confidence, thereby promoting SOC development (28). Sound “hardware” and “software” guarantees such as care facilities and institutional support, can provide a stable support environment for caregivers, reduce the sense of helplessness in the care process, and play a positive protective role in SOC (45). Other studies have shown that the SOC level of caregivers of children's families receiving standardized medical intervention gradually increases with the stability of children's condition (36). At the same time, in the investigation of parents of children with ADHD, families who received more community medical service support had better family function, which highlights the importance of professionals in community medical service (46). Conversely, inadequate access to medical care or poor-quality care can significantly undermine caregiver SOC. In areas with limited medical resources, caregivers may struggle to access timely treatment for their child, leading to increased anxiety and a sense of helplessness (47).

### 5.5 Social support factors

Social support is an important external resource to improve SOC of family caregivers of children, including emotional support, information support, material support and institutional support. Its role has been verified in multinational studies.

A growing body of research has shown that social support is an important factor in QOL and mental health of parents of children with disabilities (48–52), a lack of social support can reduce caregivers' sense of social inclusion, which in turn can exacerbate psychological anxiety among mothers of children with pervasive developmental disorders in Japan (53). In the Druze community of Israel, parents of children with developmental disabilities do not show much stress in terms of economic and psychological aspects, which may be due to the close collective culture of the Druze community. Extended families can provide economic and psychological support for parents of children with developmental disabilities to help them better cope with these pressures (54). A study on fathers of children with chronic diseases clearly pointed out that SOC of fathers was significantly positively correlated with social support, and adequate social support could enhance their understanding of life and coping confidence, thereby maintaining their mental health (47). Eriksson's systematic review found that caregivers who received adequate social support had a SOC score of 11.5 higher than those who lacked support. Emotional support could alleviate negative emotions and enhance sense of meaning in caregivers. Information support can help caregivers understand the child's disease and treatment plan, and improve the sense of understanding. Material support can reduce the financial burden of caregivers and improve their sense of manageability (9). However, a survey on parents of children with Down syndrome in Sweden showed that taking care of children may affect the career development of mothers. At this time, if society provides more opportunities for flexible employment of mothers, it will help mothers improve their sense of coherence (55). The mechanism of the factors is in Figure 1 as follows:

## 6 Interventions to improve SOC level of family caregivers of pediatric patients

### 6.1 Mindfulness therapy

Mindfulness therapy takes “non-critical awareness of present experience” as the core, and its branches include such as acceptance and commitment therapy (ACT), mindfulness-based stress reduction (MBSR), among others. By alleviating negative emotions and improving self-awareness, it can improve the level of sense of coherence (SOC) of caregivers of pediatric patients (42). Wang et al., in a study of 670 parents of children with autism, used a smartphone app to implement an 8-week mindfulness intervention (150 min per week), and the app could provide personalized exercises based on the parents' psychological state.The results showed that the anxiety and depression scores of parents were significantly reduced after intervention, and the SOC level was increased by 23%. This form of online intervention is flexible and convenient, fits the time characteristics of caregivers, and has the potential to be popularized in China. At present, mindfulness therapy (including ACT and online mode) can effectively improve the negative emotions and SOC level of caregivers of children. In the future, disease-specific intervention programs need to be explored to provide more accurate evidence-based support for clinical practice (56). This online delivery format offers several advantages: it is flexible and convenient, allowing caregivers to complete the intervention at their own pace and on their own schedule; it is cost-effective, reducing barriers to access; and it can be tailored to individual needs, enhancing engagement and effectiveness.

### 6.2 Benson's relaxation therapy

Benson Relaxation Therapy is a non-pharmacological relaxation technique proposed by Herbert Benson in 1970. Through “deep breathing, progressive muscle relaxation” and other methods, it can reduce the physiological stress response of caregivers, such as increased heart rate, increased blood pressure, and relieved anxiety, so as to improve SOC level. Mowla et al., in a study of 100 parents of children with chronic diseases in Iran, divided caregivers into an intervention group and a control group. The intervention group received Benson's relaxation therapy plus brief psychoeducation: 30 min sessions twice a week for 8 weeks. Benson's relaxation therapy includes the 4-7-8 breathing technique (4 s of inhalation, 7 s of breath-holding, and 8 s of exhalation) and the muscle relaxation training (gradual muscle relaxation from head to feet). Psychological education included disease knowledge and care skills guidance. The results showed that the SOC level of the parents in the intervention group was significantly higher than that in the control group, and the quality of life was improved by 18% (41). Another study by Molazem in 2021 further confirmed that Benson's Relaxation Therapy combined with psychoeducation can significantly improve the sense of management of caregivers—parents said that “relaxation training makes me calmer, and psychoeducation makes me master more care skills and feel more confident to meet challenges” (57).

### 6.3 Brief psychoeducational intervention (BPI)

Psychological education intervention seems to have achieved remarkable results. The researchers conducted four 60–70 min psychological education courses for the caregivers of children with chronic diseases. Each session consisted of lectures, group discussions, and relaxation techniques training. The lectures covered disease knowledge, caregiving skills, stress and anxiety management, the cultivation of positive thinking, and effective communication. At the end of the course, relevant guidance manuals on diseases and psychological intervention were distributed, and the researchers would provided regular reminders for to practice and answered participants’ questions in a timely manner. The study showed that the sense of coherence of family caregivers of children with chronic diseases was significantly improved than before.This low-cost and high-safety intervention can provide a valuable method for the multidisciplinary team to develop psychological intervention plans for caregivers (41).

### 6.4 Family resilience training

**“**Resilience” refers to the capability of families to provide additional support resources when addressing illness-related challenge. At the same time, the care and companionship provided by other family members can also help enhance the caregivers’ confidence in dealing with care-related problems, enabling them to better cultivate positive emotions and reduce the negative impact of caregiving stress on their SOC levels (58). In a randomized controlled study of improving family resilience based on a mobile device, parents of children with cancer participated in eight stress training sessions, which included detailed skill training methods with pictures or short videos, examples of applying these skills when caring for a child with cancer, and an assignment to consolidate. Participants read the content of tweets and completed web-based assignments, with each training session taking approximately 15 min. The psychological counselor would provide feedback to the participants through wechat within one working day. The psychological resilience of the children's parents was significantly improved (59). Similar results were found in the study by Park M and colleagues (60). In the future, researchers need to develop more methods to enhance psychological resilience to help family caregivers of children with chronic diseases restore their psychological balance.

## 7 Controversies and deficiencies of current research

Intervention studies remain underrepresented, and there is an notable lack of longitudinal research and qualitative research. The lack of longitudinal research makes it impossible to understand the dynamic changes of SOC of caregivers through the treatment stages of children, and it is difficult to accurately identify the key inflection points in caregiver's SOC levels. The lack of qualitative research leads to a lack of in-depth understanding of the subjective experience of caregivers, such as “how caregivers understand the sense of meaning in care” and “which resources are most effective in improving SOC”, which makes it difficult to develop targeted interventions.

In addition, the study population had notable limitations. The existing research objects mainly focus on the caregivers of children with autism, congenital heart disease, and asthma, and research on caregivers of children with rare diseases remains largely absent. Children with rare diseases have greater care pressure, and their caregivers may have a lower SOC level, and the influencing factors are different from those of caregivers of children with common chronic diseases (61). In addition, there are few studies on grandparent caregivers -grandparent caregivers generally have problems such as “older age, low education level, and insufficient care knowledge”, and their SOC characteristics are different from those of parental caregivers and need to be studied separately.

## 8 Conclusion

This review summarized factors that influence the SOC level of caregivers, including caregivers themselves, the children, the family and society. High SOC level can significantly reduce the care burden, and improve quality of life of caregivers and the health outcomes of pediatric patients. The existing measurement tools are mainly the SOC-13 Scale、the SOC-29 Scale and the SOC-L9 Scale, and the interventions are mainly mindfulness therapy、 Benson Relaxation Therapy、Brief Psychoeducational Intervention、family resilience training. In the future, it is recommended that research on SOC among family caregivers of children with chronic disease be further advanced by expanding research design,such as increasing longitudinal research and qualitative research, developing disease-specific measurement tools, focusing on special care populations, and exploring the mechanism of action in depth.
